# Case Report: Exploring the link between severe gingivitis and inflammatory bowel disease in a young patient

**DOI:** 10.3389/fdmed.2026.1744911

**Published:** 2026-05-04

**Authors:** Yuriko Maruya, Seira Hoshikawa, Momoko Fujiogi, Yuma Tsuruta, Ryoko Hino, Manami Tadano, Shinji Otake, Yuta Chiba, Aya Yamada, Satoshi Fukumoto, Kan Saito

**Affiliations:** 1 Division of Pediatric Dentistry, Department of Community Social Dentistry, Tohoku University Graduate School of Dentistry, Sendai, Japan; 2 Section of Pediatric Dentistry, Division of Oral Health, Growth and Development, Faculty of Dental Science, Kyushu University, Fukuoka, Japan

**Keywords:** Crohn’s disease (CD), dental, gastrointestinal disease, gingivitis, inflammatory bowel disease (IBD), pediatric, systemic disease

## Abstract

Severe gingival inflammation, which is uncommon in pediatric patients, often indicates underlying systemic conditions. Leukemia, autoimmune diseases, and allergic symptoms can cause gingivitis owing to systemic inflammation. Several gastrointestinal diseases, such as inflammatory bowel disease (IBD), can lead to gingival inflammation. We encountered a case of gingivitis in young child with constipation, which may be a symptom of gastrointestinal disease. The case exhibited recurrent gingival erythema and swelling accompanied by a history of constipation. Furthermore, the case exhibited perianal symptoms, such as fissures or abscesses. Upon referral to a pediatrician, blood tests were performed. The white blood cell levels were elevated, suggesting that systemic inflammation was involved. Leukemia was ruled out as a differential diagnosis. Severe gingivitis prompted a pediatrician to perform further invasive examinations, such as endoscopy in the case. Ultimately, the case was diagnosed with Crohn’s disease (CD), an IBD. These findings highlight the importance of considering systemic diseases in pediatric patients presenting with atypical or treatment-resistant gingivitis. Early recognition and interdisciplinary collaboration can facilitate prompt diagnosis of IBD and improve patient outcomes. Thus, gingival swelling may serve as a sentinel sign of systemic inflammation in children.

## 1 Introduction

Periodontal diseases encompass a variety of inflammatory conditions that affect the supporting structures of teeth, including the gingiva, periodontal ligament, and alveolar bone (1). In adults, these diseases are predominantly linked to bacterial plaque accumulation and the host’s immune responses (2–4). Even in children, bacterial plaque remains the primary etiologic factor for gingival inflammation, although this can be exacerbated by systemic conditions (5, 6). Systemic health issues can significantly alter the host immune response, making gingival tissues more vulnerable to inflammation and infection. Severe and widespread gingivitis is typically associated with conditions that lead to immunodeficiency, and infections, such as those caused by human herpes simplex virus-1 (HSV-1), which may exacerbate gingival inflammation (7–9). Autoimmune diseases, such as Behçet’s disease, asthma, allergic symptoms, and atopic disease may also cause gingivitis (10, 11). Gingival inflammation is reportedly associated with mucosal abnormalities, with inflammatory bowel disease (IBD) considered one of the causes of gingival inflammation. IBD is a chronic condition affecting the gastrointestinal tract, primarily comprising Crohn’s disease (CD) and ulcerative colitis (UC) (12). These conditions are characterized by fluctuating periods of remission and exacerbation, with symptoms ranging from mild discomfort to severe complications (13, 14). The precise etiology of IBD remains unclear, but it is considered to involve a multifactorial interplay of genetic, environmental, and immunological factors (15, 16). Specifically, CD is a granulomatous inflammatory disorder that can affect any segment of the gastrointestinal tract, presenting significant prevalence among adolescents and young adults (17). Its hallmark is transmural inflammation that can result in complications such as strictures, fistulas, and abscesses. The oral manifestations of CD are well-documented and may include oral ulcers, granulomatous cheilitis, and cobblestone mucosa (18, 19). Gingival swelling is less commonly reported; however, it can occur as a result of systemic inflammation related to the disease. Conversely, UC primarily affects the colon and rectum, resulting in continuous mucosal inflammation (20, 21). While oral manifestations in UC are generally less common than in CD, patients may still exhibit oral lesions, including pyostomatitis vegetans, which is considered a specific oral manifestation associated with UC. In our clinical practice, we encountered a case where young child presented with extensive gingival swelling. Upon assessing their oral symptoms in conjunction with their medical histories, we found a potential association with IBD. We report on a case of young child who exhibited extensive gingival swelling. This case highlights the need to consider systemic conditions such as IBD when diagnosing severe gingival inflammation in pediatric patients.

## 2 Case description

**Figure 1:**
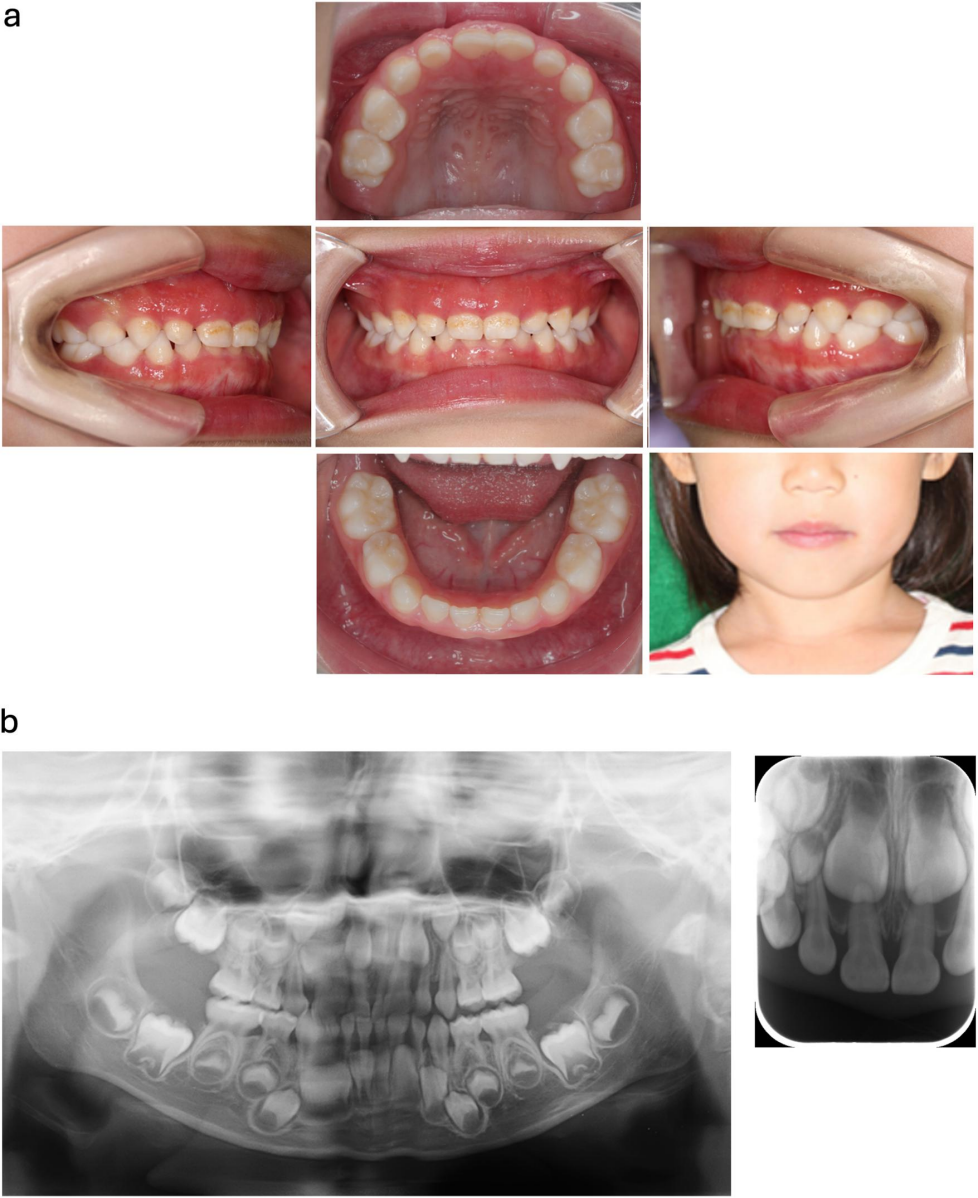
Clinical findings at the initial visit (5 years 4 months old). **(a)** Intraoral photograph showing severe, diffuse gingival erythema and swelling in both the maxillary and mandibular arches. Significant plaque accumulation is visible on the cervical areas of the deciduous teeth. **(b)** Dental and panoramic x-rays revealing no significant alveolar bone resorption, suggesting the inflammation is primarily confined to the soft tissues.

**Figure 2:**
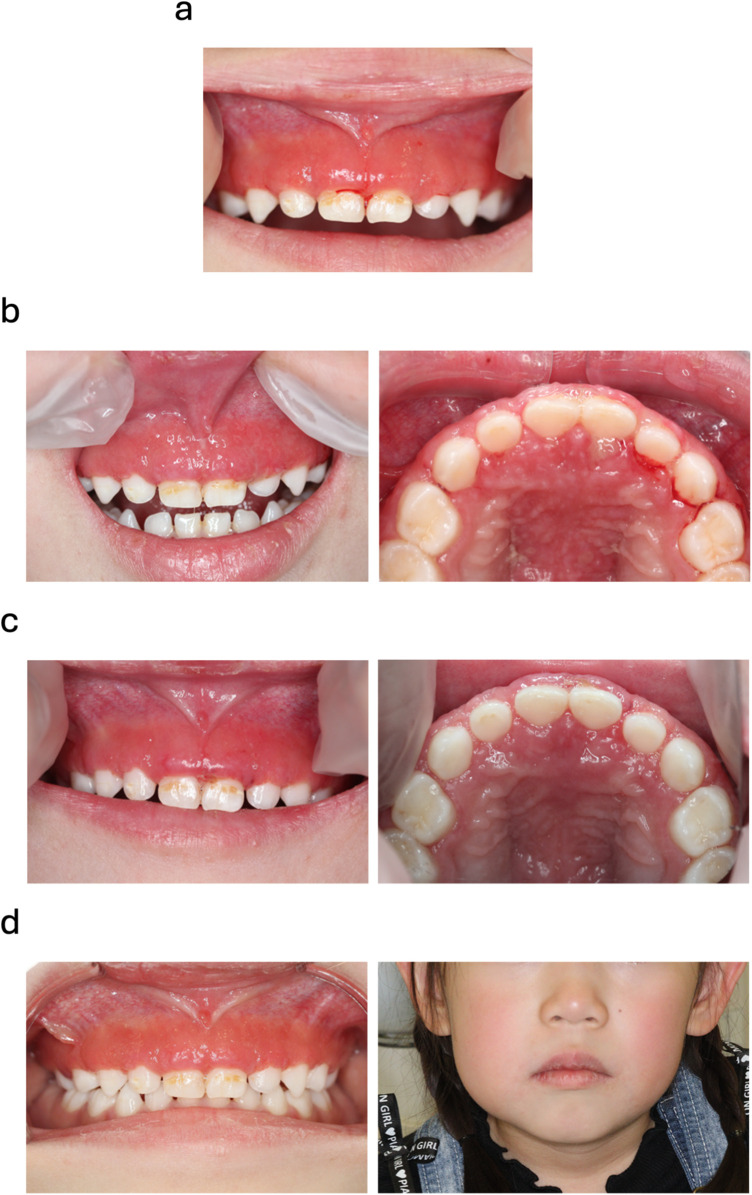
Chronological follow-up of intraoral and facial manifestations. The images show the progression of the gingival lesions despite local dental interventions. **(a)** 1month post-initial visit (5 years 5 months old): persistent gingival swelling. **(b)** 4 months post-initial visit (5 years 8 months old): worsening of the gingival enlargement. **(c)** 5 months post-initial visit (5 years 9 months old): severe, bulbous gingival proliferation. **(d)** 6 months post-initial visit (5 years 10 months old):.

A 5-year-4-month-old girl presented with gingival swelling. At 5 years 1 month old, gingival bleeding during brushing led her to visit a local dental clinic. Inflammation of the labial gingiva in the maxillary anterior region was observed. She was treated with antibiotics and received hygiene instruction, which improved her symptoms temporarily. Bleeding returned after two months, and she was referred to our department. Her medical history included asthma, atopic dermatitis, salmon roe and cat allergies, and chronic constipation. She was taking montelukast and magnesium oxide. Her father had kiwi fruit and cat allergies. Her height and weight were 105.2 cm and 16.2 kg (Kaup index, 14.64), respectively. The Kaup index [weight (g)/height (cm)² × 10] was 14.64. This index is a standard anthropometric measure used in Japan to assess the nutritional status of infants and young children, equivalent to the Body Mass Index (BMI) used in adults. In this case, it indicated that the patient’s physical development was within the normal range. Intraoral images were photographed using a digital camera and previously described standard techniques (22). Oral examination findings indicated maxillary gingival erythema and swelling, enamel caries on primary anterior teeth, lip incompetence, and mouth breathing. Radiographs showed no alveolar bone resorption (Figures 1a, b). Upon initial examination, the patient’s oral hygiene was assessed as poor, with visible plaque accumulation noted on the cervical areas of the anterior deciduous teeth. Initial treatment included brushing instruction, professional mechanical tooth cleaning and oral hygiene instructions were provided to rule out simple plaque-induced gingivitis. At 5 years 5 months old, although her oral hygiene improved, her symptoms persisted, prompting pediatric referral (Figure 2a, Table 1). Blood test results showed elevated fibrinogen and she was HSV-1 negative (Table 2). Leukemia and HSV-1 infection were denied as differential diagnoses. The pediatrician first suspected that the allergy caused gingivitis due to the rapid onset of gingival swelling and the patient’s history of hypersensitivity. A type I hypersensitivity reaction was considered a primary differential diagnosis, supported by a positive Radioallergosorbent Test for salmon roe and cat allergies and anti-allergy treatment continued. She also reported painful defecation, and anal fissures were treated with steroid ointment and macrogol (Table 1). At 5 years 6 months old, the gingival symptoms recurred. At 5 years 7 months old, perianal dermatitis appeared and she was treated with antifungal ointment. At 5 years 8 months old, the erythema had spread with ulceration (Figure 2b), but fungal test results were negative. At 5 years 9 months old, her symptoms improved (Figure 2c). At 5 years 10 months old, cheek swelling developed (Figure 2d). This case was particularly challenging because the patient lacked the typical triad of CD (diarrhea, abdominal pain, and weight loss). However, the “critical turning point” occurred when the severe gingival swelling remained completely refractory to PMTC and eventually spread to the buccal mucosa. The failure of local dental interventions over a 6-month period, combined with the emergence of anal fissures, led us to strongly suspect a systemic etiology. Following our referral, the patient was diagnosed with Crohn’s disease at a specialized pediatric gastroenterology hospital. While the definitive diagnostic procedures, such as endoscopy and biopsy, and the specific medical regimen were managed exclusively at that institution, the diagnosis was clinically confirmed and communicated to our department. Although direct access to endoscopic images or detailed medication lists was limited, the rapid and complete resolution of the gingival lesions immediately following the initiation of systemic therapy—without any change in dental protocols—serves as compelling retrospective evidence for the diagnosis and its oral manifestation. The patient has since transitioned to a local primary care dentist, remaining in complete remission for over 6 months (Table 1).

**Table 1:** Chronological summary of clinical findings, diagnostic transitions, and multidisciplinary interventions.

Age	Systemic findings	Dental/medical interventions	Clinical impression/diagnosis
4y 6m	Defecation pain, constipation. History of asthma.	Initiation of laxatives (Macrogol). Allergy testing (RAST).	Constipation, Type I Hypersensitivity.
5y 4m	First Dental Visit: Severe gingival swelling and erythema.	Started PMTC and oral hygiene instructions.	Plaque-induced gingivitis (Initial suspicion).
5y 5m	Appearance of anal fissures.	Antifungal treatment for suspected perianal infection.	Suspicion of infection (e.g., Candidiasis).
5y 10m	Persistent gingival enlargement, cheek swelling, and perianal dermatitis.	Referred to Pediatrician. Endoscopy and biopsy performed.	Crohn’s Disease (Confirmed).
Post-Diagnosis	Resolution of all symptoms.	Initiation of systemic therapy. Transition to local dental maintenance.	Remission (No recurrence for >6 months).

y, year; m, month.

**Table 2:** Blood examination results of the case reported in this study.

Laboratory parameter	Patient’s value	Reference range	Unit
White blood cell	13.3 *	3.3–8.6	10³/µL
Red blood cell	4.87	3.86–4.92	10⁶/µL
Hemoglobin	13.1	11.6–14.8	g/dL
Hematocrit	40.4	35.1–44.4	%
Mean corpuscular volume	83	83.6–98.2	fL
Mean corpuscular hemoglobin	26.9	27.5–33.2	pg
Mean corpuscular hemoglobin concentration	32.4	31.7–35.3	g/dL
Platelet	514 *	158–348	10³/µL
Fibrinogen	536 *	200–400	mg/dL
Natural killer cell	4.6	4.0–33.0	%
HSV-1 antibody titer (IgG)	0.3	< 2.0	index
HSV-1 antibody titer (IgM)	0.17	< 0.8	index

*Outside the reference range.

### 2.1 Outcome and follow-up

After achieving systemic remission, the patient was referred back to a local primary care dentist for routine maintenance, reflecting a successful transition to community-based care. We have continued to monitor the patient’s status through periodic follow-up interviews with the guardian for 6 months. We have confirmed that the patient has remained asymptomatic with no recurrence of gingival swelling or bleeding for over six months post-treatment.

## 3 Discussion

In cases of persistent gingivitis, a broad range of differential diagnoses must be considered. While plaque-induced gingivitis is the most common etiology, its lack of response to professional teeth cleaning and improved oral hygiene in this patient prompted further investigation into systemic involvements (Figure 2a). Initially, a localized allergic reaction was suspected due to the rapid onset and diffuse nature of the swelling. Other inflammatory conditions, including immune deficiencies such as leukemia or neutropenia also may be a cause of severe gingivitis in children (23–25). Furthermore, asthma, allergic symptoms, and atopic diseases cause gingivitis owing to systemic inflammation (10, 11). Additionally, severe nutritional and metabolic disorders, such as general malnutrition or vitamin C deficiency (scurvy), should be considered in the differential diagnosis, as these can predispose pediatric patients to severe atypical periodontal conditions like necrotizing ulcerative gingivitis (NUG) (26–28). The case presented in this study showed an increase in white blood cell levels, indicative of the patient developing systemic inflammation (Table 2). This highlights the need for broader differential diagnoses while evaluating gingival inflammation, especially in patients with concurrent systemic signs. As highlighted by Pecci-Lloret et al., oral manifestations such as gingivitis and oral ulcer can often be the primary or even the sole initial manifestation of Crohn’s disease, preceding intestinal symptoms (19). This underscores the critical role of dentists in identifying potential systemic diseases through careful oral examination and appropriate referral for systemic evaluation. Interdisciplinary collaboration is essential, and dental professionals must consider potential systemic diseases and communicate with pediatricians. Early detection and intervention can significantly improve long-term health. Dental professionals are pivotal in early detection, given the potential for oral manifestations to precede significant signs of systemic disease underlying non-specific symptoms, such as gingival swelling. Early recognition and referral can improve outcomes in pediatric patients with systemic diseases.

A correlation between the oral mucosa and gastrointestinal health has been proposed in the literature (29–31). The microbiomes of the oral cavity and gut have been found to show similarities. Furthermore, immune system abnormality and dysbiosis are commonly seen as the “gum–gut axis” in IBDs (30). Extraintestinal manifestations of CD have been reported at rates ranging from 6% to 53% (32). Oral findings are the most commonly observed, accounting for over half of cases. Since the average age at CD diagnosis is between 10 and 30 years, disease progression may lead to the diagnosis of IBD. Pediatric IBDs, including CD and UC, present significant diagnostic challenges owing to atypical and non-specific initial symptoms. Unlike adults, children often exhibit subtle signs, such as generalized abdominal discomfort, fatigue, anemia, weight loss, and growth failure, which precede classical gastrointestinal symptoms, complicating early diagnosis (33, 34). Growth failure, often owing to malnutrition and inflammation, can be a critical early indicator of pediatric IBD (34, 35). Perianal disease (fissures, fistulas, and abscesses) is an important marker in pediatric CD. However, such manifestations may not be immediately apparent in young patients and can be easily misattributed to benign causes, such as constipation (36–38). Our case involved a young patient with severe constipation requiring medication. Initial dental consultations revealed pronounced gingival swelling, which appeared to be localized; however, the oral symptoms suggested potential systemic involvement. Initial test findings showed minimal inflammation, and perianal fissures were linked to constipation without a clear systemic diagnosis. While the patient had poor oral hygiene and initial hygiene interventions provided temporary improvement, the gingival symptoms recurred and persisted. This refractory nature combined with the development of perianal lesions and eventual cheek swelling (Figure 2d), suggests that systemic factors, such as allergic predispositions or the underlying systemic inflammation of CD, were exacerbating the host response to plaque, collectively warranting pediatric gastroenterology referral for endoscopic examination. A limitation of this case report is the lack of specific laboratory data from the initial systemic diagnosis due to the patient being managed across different medical institutions. However, the temporal correlation between the oral treatment and the subsequent systemic diagnosis remains clinically significant.

The challenges in diagnosing pediatric IBD often necessitate invasive procedures, such as colonoscopy and endoscopy, which are crucial for disease confirmation but are seldom initiated solely on oral evidence (39–41). The possible mechanisms of IBD include multiple factors, complex microbiome-associated causes, environmental factors, and genetic abnormalities. Genes related to immunodeficiency (*NOD2/CARD15*, *OCTN*, and *TLR*), inflammation (*IL23R* and *STAT3*), and epithelial barrier homeostasis (*IBD5* and *DLG5*) have been proposed as key factors in CD prognosis (29, 42). These genetic abnormalities may contribute to gingival inflammation. This extended diagnostic process can delay interventions, emphasizing the importance of long-term oral and systemic health monitoring. The development of less invasive examination and diagnostic methods for IBD and further analysis of the pathogenesis of IBD is required in the future.

The clinical course of this case underscores that the ability to detect potential systemic diseases through the perception of oral manifestations must be recognized as a core competency for dentists. In daily practice, dentists are often the first healthcare providers to encounter patients with undiagnosed systemic conditions, such as Crohn’s disease, which may initially present solely as gingival enlargement or oral ulcerations. Identifying such correlations allows for early diagnosis and ensures that patients receive timely, life-saving medical treatment. Therefore, it is imperative that dental education places greater emphasis on developing this diagnostic acumen, training future dentists to look beyond the oral cavity and consider the patient’s systemic well-being as a fundamental part of their professional responsibility.

## 4 Patient perspective (from the patient’s guardian)

The patient’s guardians expressed deep relief when the underlying cause was finally identified, as the persistent oral symptoms had been a source of significant concern. They shared their hope that this case would be reported to help other clinicians recognize the link between oral health and systemic diseases, potentially leading to earlier diagnoses for other children in similar situations.

## Data Availability

The original contributions presented in the study are included in the article/supplementary material, further inquiries can be directed to the corresponding author.
